# Infected congenital cervical dermal sinuses leading to spinal cord abscess: two case reports and a review of the literature

**DOI:** 10.1007/s00381-020-04778-1

**Published:** 2020-07-06

**Authors:** Rhian Bevan, Paul Leach

**Affiliations:** 1 University Hospital of Wales College of Medicine, Cardiff, UK; 2 Department of Paediatric Neurosurgery, University Hospital of Wales, Cardiff, UK

**Keywords:** Dermal sinus, Cervical, Intramedullary abscess, Spinal dysraphism

## Abstract

**Purpose:**

Congenital dermal sinuses are a rare form of spinal dysraphism. The developmental defects are located along the midline neuroaxis, with sinuses in the cervical region being the least common. Congenital dermal sinuses can be associated with intraspinal infection as they act as a direct route from the skin and subcutaneous tissues into the spinal cord.

**Methods:**

The authors present two cases of cervical dermal sinuses complicated by intramedullary abscess. Both children presented with neurological decline and febrile illness. MRI showed intraspinal abscess. Both underwent prompt surgical excision of the sinus tract, exploration of the cord and intravenous antibiotics.

**Results:**

Both patients demonstrated excellent neurological recovery.

**Conclusions:**

Complete surgical excision of the sinus and tract in addition to long-term antimicrobials can yield excellent neurological outcomes. At surgery, do not expect to find pus when exploring the intramedullary component. Long-term follow-up is advocated due to potential late recurrence.

## Introduction

Congenital dermal sinuses are a rare form of spinal dysraphism occurring in approximately 1 in 2500 live births.^2^ The structural malformation results from the incomplete separation of ectoderm and neural ectoderm during development, leading to a cutaneous fistula that can extend from subcutaneous tissues down to the spinal cord.^4^ This direct communication is a potential route for the spread of infection to the central nervous system which may result in meningitis or intradural abscess. The association between intradural abscess and dermal sinus tracts are infrequent; however, without early diagnosis and treatment, irreversible spinal cord damage can result.

Dermal sinus tracts may occur along the midline neuroaxis, but only 1% of all tracts are in the cervical region.^5^ There is limited literature detailing the management of cervical intradural abscesses in association with congenital dermal sinuses, and we present two cases of cervical congenital dermal sinuses that presented with an intraspinal abscess.

## Case 1

A 3-year-old girl was admitted with gait disturbance. Physical examination showed an excoriated midline cervical dermal sinus over the C6 spinous process. The child had a wide-based, hypertonic gait with hyperreflexia in the lower limbs bilaterally. Magnetic resonance imaging (MRI) of the spine showed an enhancing intradural lesion at C5/C6 which communicated with the subcutaneous tissues posteriorly. This was suggestive of an intradural abscess resulting in a swollen cord with oedematous changes extending from the medulla down to T9.

**Fig. 1:**
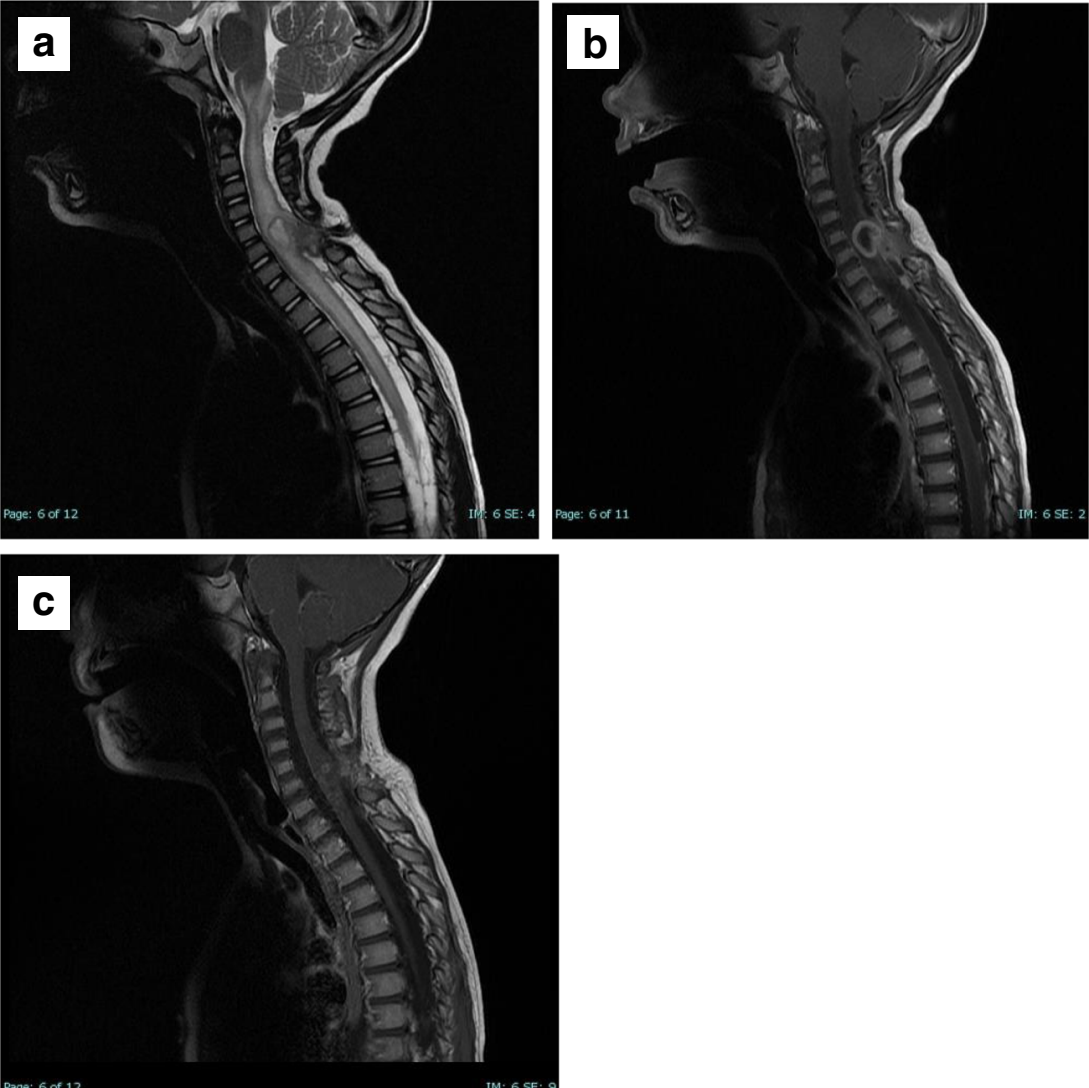
Preoperative sagittal MRI of the spine. **a** T2-weighted image demonstrating a dermal sinus tract at C6, connecting the skin to spinal cord and associated oedema extending from the medulla to T9. **b** Preoperative T1-weighted MRI with contrast showing an abscess at C5-C6. **c** Post-operative T1-weighted MRI with contrast at 6 months showing complete removal of dermal sinus tract and resolution of the abscess.

The child underwent surgical excision of the dermal sinus. The tract was dissected down to the dura where the wall of the sinus was in continuity with the spinal dura. No pus was encountered within the cord, only inflamed-looking dermoid-type tissue. Samples were sent for histopathological and microbiological examination. It revealed an epithelial-lined cyst, fibro-fatty connective tissue and skeletal muscle fibres with inflammatory cell infiltration, confirming the diagnosis of dermal sinus. There was no growth on bacterial culture. The child received 6 weeks of intravenous and 4 weeks of oral antimicrobials. Clinical examination at 6 months demonstrated a complete neurological recovery. There has been no recurrence to date after 9 years of follow-up Fig 1.

## Case 2

A 16-month-old girl presented with rapid onset neck swelling, torticollis and lethargy. On examination, temperature was 38.2 °C, and she was tachycardiac and tachypnoeic with an increased work of breathing for which she needed intubation and ventilation. On inspection, there was a midline birthmark with a pinhole opening in the mid cervical region. MRI of the spine showed an intraspinal abscess originating from a dermal sinus tract at C4 with oedema extending from the pons to T1.

**Fig. 2:**
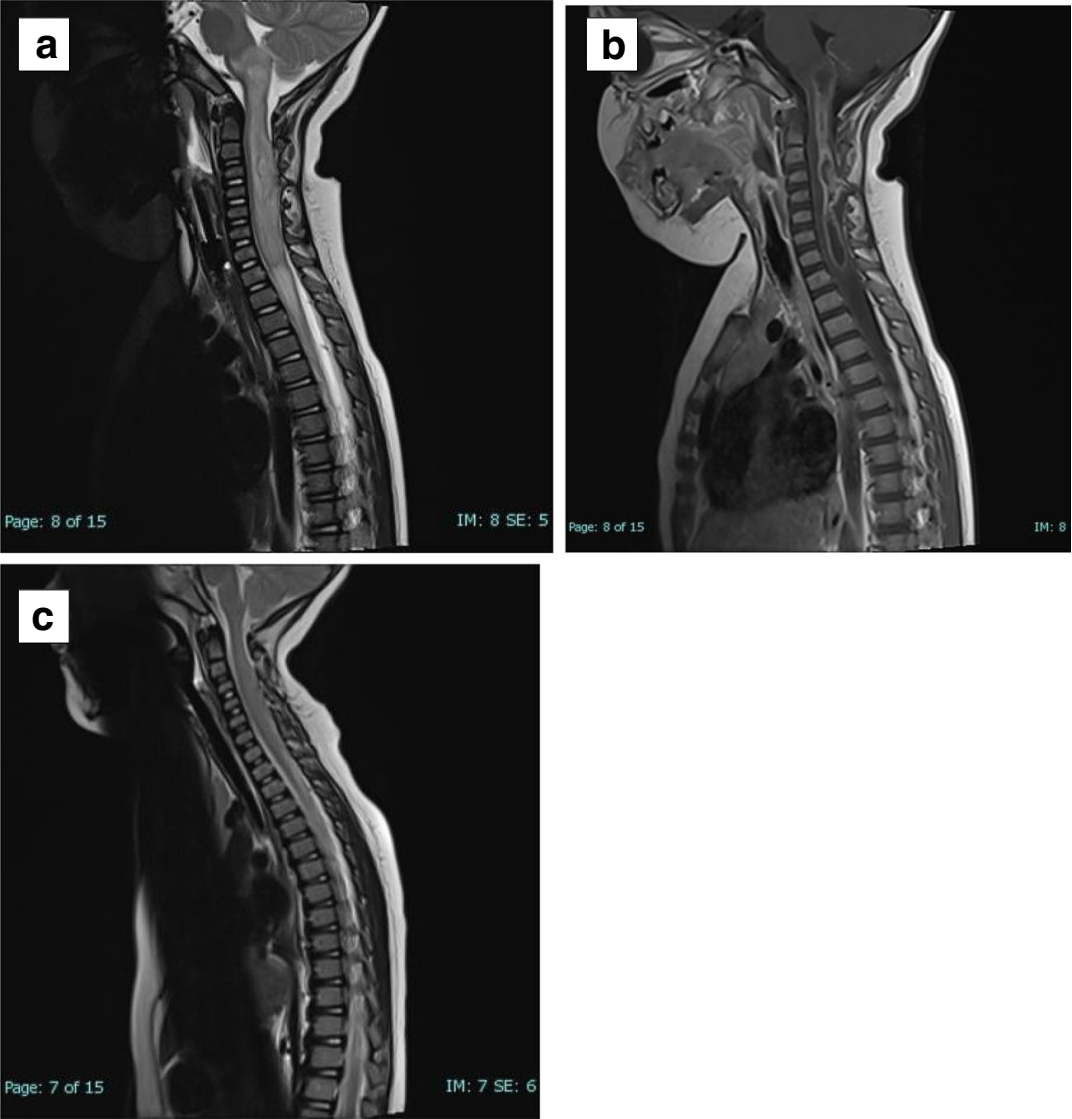
Preoperative sagittal MRI of the spine.: (**a**) T2-weighted image showing oedema of the pons extending down to T1 with an associated intradural lesion at C4.. (**b**) Preoperative T1-weighted MRI with contrast showing an extensive intramedullary abscess. (**c**) Post-operative T2-weighted MRI of the spine at 9 months showing resolution of the abscess and oedema

The child was taken to theatre for exploration of the spinal abscess, and the dermal sinus was excised, and inflamed-looking intramedullary dermoid tissue was biopsied. No intramedullary pus was found. Tissue pathology revealed an epithelial-lined cyst with a central punctum and keratinous debris with polymorph infiltrate in keeping with a dermal sinus and intramedullary dermoid tissue. Bacterial culture grew *Propionibacterium* species. Post-operatively her left arm was plegic. She received 6 weeks of intravenous antimicrobials and made a full neurological recovery. At 14 months follow-up, she remains well. Fig 2.

## Discussion

Congenital dermal sinuses are persistent epithelium-lined tracts that connect the skin surface to deeper structures extending down to variable depths.^10^ The persistent connection acts as a potential conduit for the spread of infection down into deeper neural structures, presenting with meningitis or less commonly as epidural, subdural or intramedullary abscesses.^14^

Historically, the first presentation of a congenital dermal sinus was following an infection of the central nervous system, presenting through rapid neurological decline from inflammation or mass effect.^12^ However, congenital dermal sinuses are now more frequently being found by primary care physicians on physical examination. Alongside identifying a presumed sinus opening, other cutaneous findings such as dimples, pigmented changes and abnormal hair growth are associated with congenital dermal sinuses.^1^, ^6^ An important differential diagnosis for the cutaneous markers seen in dermal sinuses is the skin lesions seen in limited dorsal myeloschisis as described by Pang et al. .^11^ Congenital dermal sinuses can be located anywhere along the midline neuroaxis, with approximately 90% located in the lumbosacral region, 10% in the thoracic region and 1% found at the cervical level.^5^ In our two case reports, the lesions were located in the cervical area, and both patients presented with neurological decline and febrile illness. MRI whole spine is the gold standard investigation for rapid identification of dermal sinuses and associated abscesses. Additionally, MRI is also useful in identifying potential coexisting congenital abnormalities and for surgical planning.

Once diagnosed, prompt surgical excision of the sinus tract with exploration of the abscess should be performed. This should include excision of the tract down to the dura and liberal wash out of the abscess.^7^ The authors note that in their experience one is unlikely to find pus within the cord instead solid inflammatory tissue may be encountered and removed/biopsied. Extensive dissection looking for pus may endanger the spinal cord and is not necessary to prevent recurrence.^7^

Alongside surgical management, long-term appropriate intravenous antimicrobials should be commenced.^12^ The skin commensal *Staphylococcus aureus* is the most commonly cultured organism from intramedullary spinal cord abscesses resulting from congenital dermal sinuses^15^; however, a wide range of organisms have been reported previously^4^, ^13^ The culture in one of our cases grew *Propionibacterium* species, an organism rarely found in association with an intramedullary abscesses, documented only once previously in the literature.^16^ Occasionally, cultures can be negative.^3^

Both of our patients suffered neurological deficits as a result of cervical congenital dermal sinuses complicated by infection. The need for prompt surgical intervention seems reasonable in these cases; however, the management of congenital dermal sinuses identified through cutaneous findings in routine examinations is unclear. Many authors advocate prophylactic surgical resection with complete removal of the sinus to prevent delayed presentations which are associated with worse outcomes.^1^ The literature supporting this is not tailored to cervical congenital dermal sinuses but addresses the management of more common lumbosacral congenital dermal sinuses.^8^, ^9^, ^12^ More evidence pertaining to the prophylactic removal of cervical congenital dermal sinuses is required to assess the benefits of surgery against the risks, such as infection. There is however clear consensus in the literature that suspected congenital dermal sinuses should be further investigated with MRI to establish the full extent of the tract and any associated pathology.^1^

## Conclusion

Both of our case studies presented with intradural abscesses secondary to cervical dermal sinus tracts. We advocate emergency excision of the sinus and tract down to dura and exploration of the cord abscess; however, pus may not be encountered, and extensive cord dissection should be avoided. The role of prophylactic removal of the sinus tract in the cervical region is currently undocumented in the literature.
